# Multisystem Infantile Hemangiomatosis with Cutaneous, Hepatic, and Splenic Involvement

**DOI:** 10.3390/pediatric17050102

**Published:** 2025-10-03

**Authors:** Elvira Ioana Buda, Alina Grama, Mădălina Bota, Alexandra Mititelu, Gabriel Bența, Diana Borcău, Otilia Fufezan, Cristina Blag, Tudor Lucian Pop

**Affiliations:** 1 2nd Pediatric Discipline, Department of Mother and Child, “Iuliu Hațieganu” University of Medicine and Pharmacy, 400012 Cluj-Napoca, Romania; buda_elvira_ioana@elearn.umfcluj.ro (E.I.B.); madalina.bota@umfcluj.ro (M.B.); perta.alexandra@elearn.umfcluj.ro (A.M.); benta.gabriel@elearn.umfcluj.ro (G.B.); dianaborcau@gmail.com (D.B.); lucia.blag@elearn.umfcluj.ro (C.B.); tudor.pop@umfcluj.ro (T.L.P.); 2 2nd Pediatric Clinic, Emergency Clinical Hospital for Children, 400177 Cluj-Napoca, Romania; 3 Imaging Department, Emergency Clinical Hospital for Children, 400370 Cluj-Napoca, Romania; otilia.fufezan@gmail.com

**Keywords:** hemangiomatosis, neonates, visceral hemangiomas, cholestasis, propranolol

## Abstract

Background: Hemangiomatosis is a rare condition characterized by the presence of multiple benign vascular tumors that may affect various organs, including the skin, liver, and spleen. Complications are closely linked to the location and size of the lesions. Case Presentation: We describe a rare presentation of infantile hemangiomatosis with widespread cutaneous and oral mucosal lesions, further complicated by splenic and hepatic involvement and secondary cholestasis. The initial progression was unfavorable, with an increase in both the number and size of the lesions. Cardiologic evaluation identified minor valvular insufficiencies, but no secondary cardiac failure. Treatment with propranolol and prednisone was initiated, with a slow favorable evolution. There were no new hemangiomas developed, and those on the face and limbs decreased in size, some disappearing entirely. Hepatic and splenic hemangiomas regressed more slowly, but their reduction and the improvement of cholestasis were progressive. Due to significant iatrogenic Cushing’s syndrome, prednisone was gradually tapered. Transient subclinical hypothyroidism occurred during treatment, resolving spontaneously. Conclusions: The present case illustrates the rarity and complexity of multifocal infantile haemangiomatosis and highlights the importance of early diagnosis, comprehensive organ evaluation, and tailored multidisciplinary management. It clearly demonstrates that prompt intervention and careful therapy adjustment can lead to favorable outcomes even in the setting of extensive visceral involvement.

## 1. Introduction

Infantile hemangiomas (IHs) are the most common benign tumors of early childhood and result from abnormal endothelial cell proliferation. Several hypotheses regarding etiology exist, but the most probable underlying cause is hypoxic stress, which promotes the upregulation of glucose transport protein-1 (GLUT1) and vascular endothelial growth factor (VEGF) expression, subsequently triggering the mobilization of endothelial progenitor cells [1].

IHs typically appear on the head and neck and can present as focal, multifocal, segmental, or indeterminate lesions. Multifocal lesions are particularly important due to their potential for extensive involvement. Segmental IHs, the least the common type, can also be part of vascular syndromes such as PHACE(S) and LUMBAR. Awareness regarding these syndromes is crucial because they involve large segmental hemangiomas associated with congenital anomalies, requiring early diagnosis. PHACE(S) syndrome is characterized by large facial hemangiomas and multiple congenital anomalies, including neurovascular defects. LUMBAR syndrome involves large hemangiomas in the lumbosacral area, accompanied by urogenital, spinal, and other systemic malformations [2].

Multifocal infantile haemangiomatosis (MIH) is a rare and potentially life-threatening vascular disorder characterized by the presence of numerous IHs affecting the skin and a minimum of three internal organs [1]. Most commonly, the affected viscera are the liver, gastrointestinal tract, lungs, and, less frequently, the brain. This condition typically manifests at birth or within the first few days to weeks of life [3,4].

While cutaneous IHs are often benign and may undergo spontaneous involution, the involvement of visceral organs can lead to serious complications. As a result, for infants who present with five or more cutaneous IHs, a routine abdominal ultrasound is advised to screen for possible internal (particularly hepatic) involvement, allowing for timely diagnosis and intervention if necessary [5].

## 2. Case Presentation

We present the case of a female neonate, born at 41 weeks of gestation via cesarean section after a pregnancy marked by a urinary tract infection in the first trimester and vaginitis in the third. The newborn had intrauterine growth restriction, with a birth weight of only 2620 g. At birth, multiple violaceous, non-blanching maculopapular lesions were observed diffusely on the skin and the oral mucosa, consistent with hemangiomas. Over the following days, the lesions increased in number and size.

**Figure 1:**
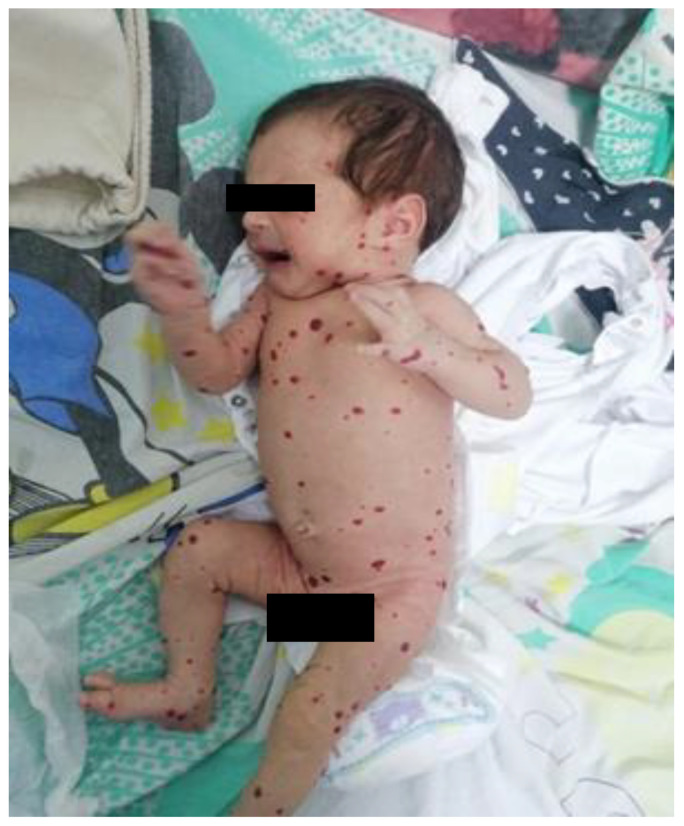
Clinical aspect—multiple cutaneous hemangiomas scattered across the entire trunk, extremities, and face, presenting as well-defined, red to violaceous papules and nodules of varying size.

**Figure 2:**
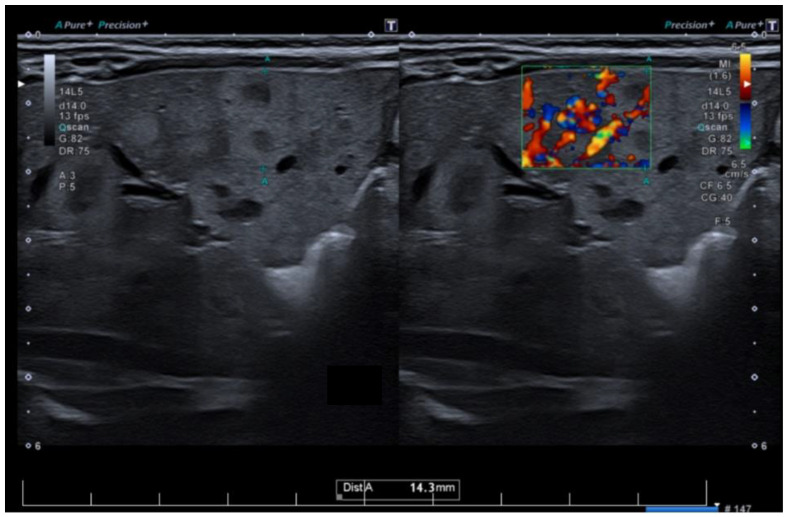
Hepatic ultrasonography with color Doppler demonstrates multiple hypoechoic lesions consistent with hepatic hemangiomas, showing internal vascular flow.

The patient was admitted to our service at two weeks of life for further evaluation. The physical examination revealed disseminated hemangiomas and subtle jaundice. Given the extensive cutaneous and mucosal hemangiomas, as illustrated in Figure 1, an abdominal ultrasound was performed. The examination revealed multiple hepatic hemangiomas (Figure 2), the largest of which measured 25 mm. Additionally, there was evidence of mild dilation and distortion of the hepatic veins, along with an enlarged hepatic artery measuring up to 4 mm. Two vascular lesions were also identified in the spleen. Doppler ultrasound showed no signs of arteriovenous shunting. Furthermore, cranial ultrasound revealed subependymal and frontal horn cysts, considered to be sequelae of a probable antenatal intraventricular hemorrhage.

Blood tests (Table 1) revealed a cholestatic hepatitis pattern, with serum gamma-glutamyl transferase (GGT) levels at 549 U/L (normal 9–39 U/L), alkaline phosphatase at 598 U/L (normal 75–316 U/L), total bilirubin at 14.97 mg/dL (normal 0.10–1.20 mg/dL), and direct bilirubin at 2.39 mg/dL (normal <0.30 mg/dL). Alpha-fetoprotein levels were markedly elevated (>1004 IU/mL); however, this was considered appropriate for the patient’s age, as it is physiologically elevated during early infancy. Despite these findings, serum aspartate aminotransferase (AST, 46 U/L; normal 9–80 U/L) and alanine aminotransferase (ALT, 12 U/L; normal 8–32 U/L) were within the normal range. Serologic tests for common perinatal infections, including Epstein–Barr virus (EBV), Cytomegalovirus (CMV), Toxoplasma, hepatitis B virus (HBV), and Herpes simplex virus (HSV), were negative.

Given that one of the most serious complications of multifocal infantile hemangiomatosis is high-output cardiac failure and considering the observed dilation of the hepatic veins on abdominal ultrasound, which raised concerns about possible cardiac involvement, further evaluation was warranted. A cardiac ultrasound was performed, revealing minor mitral, aortic, and tricuspid regurgitation, a small atrial septal defect with left-to-right shunting, and a persistent ductus arteriosus, all in the absence of clinical signs of heart failure. NT-proBNP was elevated (11,094 pg/mL; normal < 300 pg/mL), possibly reflecting the hemodynamic burden associated with visceral hemangiomas.

Propranolol treatment was started at the age of two weeks, titrated up over two weeks, and maintained at 2.5 mg/kg/day, in combination with prednisone at a dose of up to 2 mg/kg/day. Ursodeoxycholic acid was added to improve bile flow, limit hepatocellular injury, and mitigate complications of persistent cholestasis (e.g., malabsorption of fat-soluble vitamins), thereby providing supportive hepatoprotective therapy until resolution of the obstruction.

**Figure 3:**
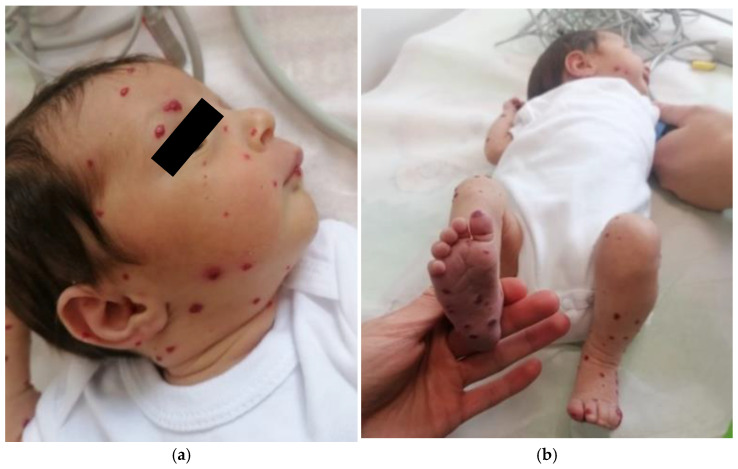
(**a**,**b**) Cutaneous hemangiomas in evolution, showing decreased size, flattening of the lesions, and fading of erythematous coloration compared with the initial presentation.

Clinical and imaging follow-up revealed a gradual regression of cutaneous hemangiomas (Figure 3a,b), particularly on the face, limbs, and thorax, as well as partial regression of hepatic and splenic hemangiomas. The cholestasis syndrome also improved progressively. However, the patient developed iatrogenic Cushing’s syndrome at the age of 6 months, leading to a progressive taper and eventual discontinuation of prednisone. During the follow-up, a transient subclinical hypothyroidism was noted, but it resolved spontaneously.

**Figure 4:**
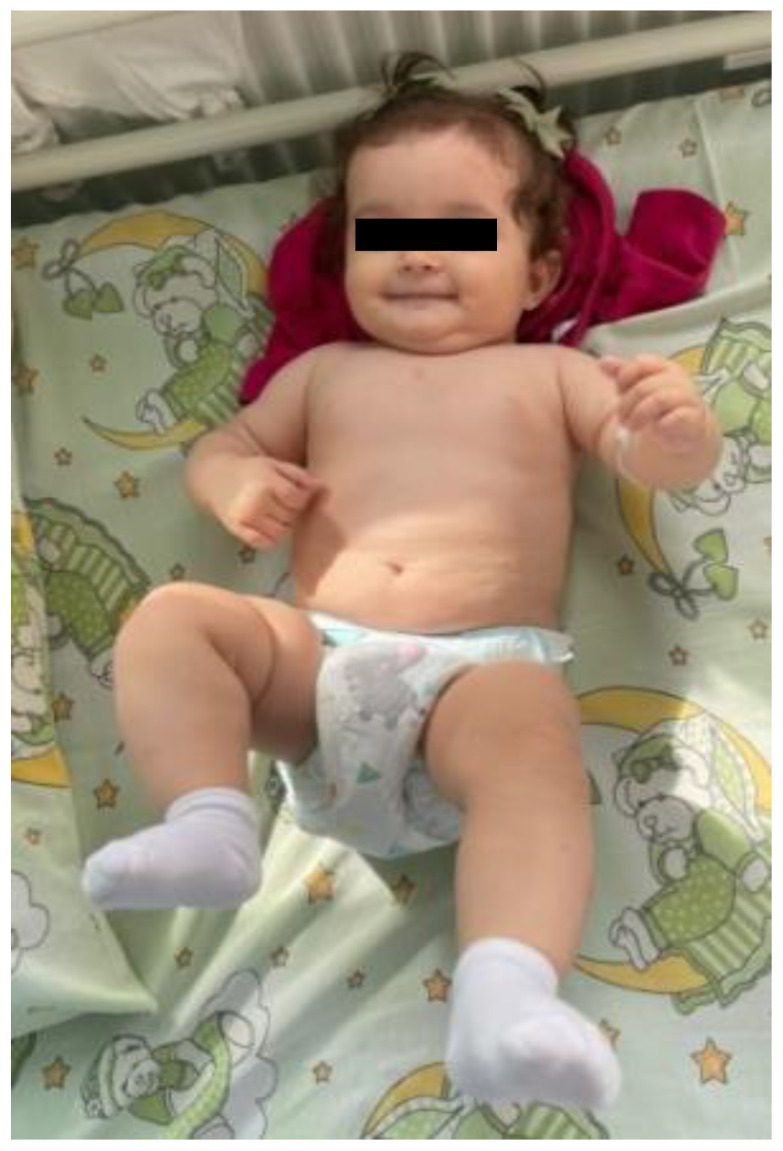
Clinical appearance of the patient after 9 months of therapy, demonstrating near-complete involution of cutaneous hemangiomas. The lesions are almost completely resolved, appearing flat, pale, and barely discernible.

After 9 months of treatment, imaging examination showed significant remission of cutaneous hemangiomas (only five residuals, smaller and paler lesions), and reduced size of hepatic and splenic lesions (Figure 4). Given the resolution of cholestasis, ursodeoxycholic acid therapy was withdrawn. Propranolol was continued at a weight-adjusted dose of 1.7 mg/kg/day. The patient remained clinically stable and was under periodic clinical, echocardiographic, hepatic, and thyroid monitoring.

## 3. Discussion

Infantile hemangiomas are among the most common tumors in infancy, with an estimated incidence ranging from 3% to 10%. Recent data suggest that the incidence has been steadily increasing over the past three decades. A longitudinal, population-based study demonstrated a significant correlation between this rising trend and factors such as decreasing gestational age and low birth weight, indicating a higher susceptibility in preterm and growth-restricted infants [6].

MIH is a rare and severe variant characterized by multiple cutaneous and visceral hemangiomas, most commonly affecting the liver, but also the spleen, lungs, and brain [7]. MIH typically occurs in patients with five or more cutaneous lesions. They are usually asymptomatic but can become clinically significant in cases of vascular shunting or diffuse liver involvement, causing massive hepatomegaly [2]. Hepatic involvement can lead to complications like high-output cardiac failure, hypothyroidism, and cholestasis [8,9].

Genetic predisposition and intrauterine environmental factors may contribute to abnormal angiogenesis, leading to the subsequent development of widespread cutaneous and visceral hemangiomas. The placental origin theory proposes that IH originates from endothelial progenitor cells (EPCs) that develop in the placenta, under the influence of both intrinsic and extrinsic factors. Intrinsic factors include dysregulation of angiogenesis, which is supported by the presence of angiogenic growth factors. Extrinsic factors, on the other hand, include hypoxia and disturbances in developmental fields. Several different factors have been associated with an increased risk of developing MHI, like female sex, low birth weight, prematurity, premature rupture of membranes, abnormal amniotic fluid volume, placenta previa, multiple gestation pregnancies, maternal exposure to progesterone during pregnancy, anemia in pregnancy, miscarriage history, family history of hemangiomas, and Caucasian heritage [10,11].

In most cases, diagnosis relies predominantly on clinical evaluation and patient history. If the diagnosis is inconclusive, if more than five lesions are present, and when an extracutaneous involvement is suspected, a comprehensive diagnostic workup should include imaging studies, with a focus on abdominal ultrasonography (US), which reveals a well-defined mass. Infantile hepatic hemangiomas are generally characterized by low or even absent intralesional Doppler signal, consistent with their slow circulation [2,3]. Increased vascularity is more commonly observed in the feeding and draining vessels surrounding the lesion, rather than within the lesion parenchyma. This distinction is clinically relevant, as it helps differentiate infantile hemangiomas from vascular malformations, particularly arteriovenous malformations, which are true high-flow entities [4]. In cases with suspected multi-organ involvement or inconclusive findings, advanced imaging techniques such as magnetic resonance imaging (MRI) or computer tomography scans (CT) may be necessary to assess the extent of visceral disease [12,13]. Histopathological confirmation using the endothelial marker GLUT-1 immunostaining is a highly sensitive and specific diagnostic tool for infantile hemangiomas. Nevertheless, liver biopsy is infrequently required in routine clinical practice and should not be performed routinely. Indications for biopsy extend beyond suspicion of malignancy and include situations such as atypical imaging findings, uncertain diagnosis, or inadequate response to standard therapy. Recently, electronic colorimeters—widely accessible on computers and mobile devices—have been increasingly used to help differentiate IHs from other vascular tumors and malformations by providing objective color analysis [2,14]. Laboratory testing plays a crucial role in assessing and monitoring MIH. Basic blood tests should include a complete blood count (to evaluate for anemia or thrombocytopenia), liver function tests (to determine the hepatic involvement and potential dysfunction), and coagulation studies. In the present case, cholestasis is primarily attributed to extrinsic compression of the intrahepatic biliary ducts by multiple hepatic hemangiomas, leading to impaired bile flow.

MIH has been known to induce high-output cardiac failure, which is why B-type natriuretic peptide should be determined. Additionally, thyroid function (TSH, free T4) is also recommended, as large hepatic hemangiomas can produce type 3 deiodinase, which inactivates circulating thyroid hormones, leading to a condition known as consumptive hypothyroidism [15,16]. Serum levels of vascular endothelial growth factor (VEGF) and basic fibroblast growth factor (bFGF) have been proposed as a tool to evaluate proliferative activity, monitor disease progression, and inform therapeutic decisions [17,18].

The differential diagnosis of IHs is crucial due to the diverse range of vascular tumors and malformations that share overlapping clinical features. IHs are characterized by a well-defined proliferative phase occurring shortly after birth, presenting as telangiectasias or reddened macules that become more visible at 2–3 weeks of age and are followed by spontaneous involution. Between 3 weeks and two months of age, IHs reach approximately 80% of their final size. In contrast, vascular malformations are congenital anomalies present at birth, growing in proportion to the child and classified according to the type of vessel involved (capillary, venous, lymphatic, arteriovenous, or mixed) and their associated hemodynamic properties. Congenital hemangiomas differ from IHs as they are fully developed at birth, do not proliferate postnatally, and lack GLUT-1 expression. Kaposiform hemangioendotheliomas and tufted angiomas are locally aggressive vascular tumors that may present in infancy or early childhood, carrying the risk of the Kasabach–Merritt phenomenon. Pyogenic granulomas, although less common in infancy, typically manifest as rapidly growing, friable lesions that are prone to bleeding. Capillary malformations tend to evolve in color and texture over time, venous malformations become apparent later in childhood, and lymphatic malformations may cause progressive functional impairment. Accurate differentiation among these entities is crucial for appropriate management and prognosis [2].

Recognizing and screening complications in MIH is a key aspect of patient management. Life-threatening complications include lesions of the lower face, due to potential airway obstruction, and the presence of five or more cutaneous IHs, which may signal hepatic hemangiomas. Functional impairment can result from periocular involvement, with risk of visual compromise, or from lesions affecting the lips and oral cavity, which may hinder feeding. The extensive nature of the vascular lesions can result in significant cosmetic and psychological impacts, especially if the condition persists beyond infancy. Ulceration occurs in approximately 16% of cases, particularly in high-risk anatomical sites (neck, intertriginous areas, lips, extremities) and in segmental subtypes, leading to secondary infections and potential impairment of growth and development in infants.

Identifying hepatic involvement at an early stage is crucial, as it allows for timely intervention and helps prevent potentially serious complications, such as high-output cardiac failure, respiratory distress, consumptive coagulopathy, and multi-organ dysfunction [5,19].

In uncomplicated cases, no pharmacological intervention is required, and active clinical surveillance is sufficient. Early specialist referral is recommended for infants with multiple lesions, high-risk hemangiomas, or those associated with syndromic presentations. The primary goal in the management of infantile hemangiomas is to prevent ulceration and life-threatening complications. Initiating treatment within the first 4–6 weeks of life can significantly reduce or even prevent complications. Management options range from pharmacological therapies (topical or systemic) to laser treatment and, in select cases, surgical intervention [20].

Considering the severity of liver involvement, our patient received prolonged treatment with propranolol in increasing doses up to 2.5 mg/kg/day combined with prednisone up to 2 mg/kg/day. Beta-blockers represent the first-line therapy, offering high efficacy in achieving lesion regression and preventing complications. Propranolol, a non-selective beta-blocker, is believed to exert phase-dependent effects in IHs, inducing vasoconstriction during the proliferative phase and reducing angiogenic factors while promoting apoptosis of capillary endothelial cells during the involution phase, ultimately leading to lesion regression. Recent guidelines recommend initiating treatment for patients under 8 weeks of age. Although generally well-tolerated, the use of propranolol can give rise to specific adverse reactions such as bradycardia, wheezing, hypotension, and hypoglycemia. Drowsiness, agitation, and sleep disturbances are usually transient effects and rarely require intervention. Atenolol is an alternative therapy due to its lower central nervous system effects and equivalent efficiency. Beta-blockers are contraindicated in asthma or bronchospasm due to the risk of airway hyperreactivity, which may also occur during respiratory infections in patients without a prior history of airway disease. It is essential to highlight that hypoglycemia is not dose-dependent, and it tends to occur in infants under 1 year of age and during periods of fasting. Treatment starts with oral propranolol given immediately after meals to minimize hypoglycemia risk. The initial dose is 1 mg/kg/day, administered in two divided doses. The dose is increased to 2 mg/kg/day after one week, followed by a further increase to 3 mg/kg/day as needed, based on the severity of the hemangioma and clinical response [2,20,21]. Rebound growth following propranolol discontinuation occurs in approximately 14–25% of children, with risk factors including younger age at cessation, presence of a deep hemangioma component, female sex, segmental distribution, and greater lesion depth. Relapses may be presented within weeks to months after treatment cessation, and while mild recurrences often resolve without intervention, more significant regrowth may require a second course of propranolol or topical beta-blocker therapy [22].

Systemic corticosteroids have traditionally been considered the first-line therapy. Still, their efficacy is inconsistent, and their use in neonates is associated with significant adverse effects (Cushing syndrome, infections, growth retardation, hypertension, mood changes, and skin complications). Oral prednisone or prednisolone in a dose of 2–3 mg/kg/day should be prescribed in cases where propranolol is contraindicated or where there is unresponsiveness to beta-blockers [12].

In refractory or life-threatening cases, various chemotherapeutic agents have been used as second-line treatments. Sirolimus demonstrates clinical efficacy in hemangiomas and complex vascular anomalies by reducing lesion size and improving symptoms. Adverse effects, including mucositis, rash, gastrointestinal disturbances, cytopenia, and metabolic alterations (such as hyperlipidemia and hyperglycemia), are generally mild and manageable with supportive care or dose adjustments. New therapeutic options include mTOR inhibitors (sirolimus) or interferon-alpha-2a. Interferon-alpha-2a has demonstrated its antiangiogenic effectiveness through the inhibition of endothelial and fibroblast proliferation. However, its use is limited by the risk of severe neurotoxicity, including irreversible spastic diplegia, necessitating close neurological monitoring. Sirolimus represents a promising alternative to conventional treatment for patients with unresectable vascular hemangiomas, but further studies are needed to validate its long-term safety [23]. In selected cases, interventional approaches like selective hepatic artery embolization or laser therapy have also been reported [21].

In our patient, prolonged corticosteroid use led to iatrogenic Cushing’s syndrome, necessitating gradual tapering and discontinuation of therapy. This highlights the importance of monitoring adverse effects and adjusting treatment regimens accordingly.

## 4. Conclusions

This case of multi-organ hemangiomatosis is notable for several reasons. The presence of multiple disseminated hemangiomas affecting the skin, liver, and spleen is rare and associated with significant risks of complications, including cholestasis and minor cardiac valvular insufficiencies. The patient exhibited a slow but favorable response to a combination of propranolol and prednisone, highlighting the need for a tailored therapeutic approach in complex cases. The slower regression of hepatic and splenic hemangiomas compared to cutaneous lesions underscores the challenges in treating visceral hemangiomas, necessitating ongoing monitoring and adjustments in therapy. Overall, this case highlights the rarity and complexity of multifocal infantile hemangiomatosis, underscoring the importance of early diagnosis, comprehensive organ evaluation, and multidisciplinary management. It demonstrates that prompt intervention and careful therapy adjustment can lead to favorable outcomes even in the setting of extensive visceral involvement.

## Figures and Tables

**Table 1:** Laboratory findings at presentation.

Test	Patient Value	Reference Range
Gamma-glutamyl transferase (GGT)	549 U/L	9–39 U/L
Alkaline phosphatase (ALP)	598 U/L	75–316 U/L
Total bilirubin	14.97 mg/dL	0.10–1.20 mg/dL
Direct bilirubin	2.39 mg/dL	<0.30 mg/dL
Aspartate aminotransferase (AST)	46 U/L	9–80 U/L
Alanine aminotransferase (ALT)	12 U/L	8–32 U/L
Alpha-fetoprotein (AFP)	>1004 IU/mL	Physiologically elevated in early infancy
NT-proBNP	11,094 pg/mL	<300 pg/mL

Note: Selected laboratory tests relevant to the patient’s clinical presentation are shown. Other routine blood tests were within normal limits.

## Data Availability

The original contributions presented in this study are included in the article. Further inquiries can be directed to the corresponding author.

## Abbreviations

The following abbreviations are used in this manuscript:

IH	Infantile Hemangiomatosis
GLUT 1	Glucose Transport Protein-1
VEGF	Vascular Endothelial Growth Factor
MIH	Multifocal Infantile Hemangiomatosis
US	Ultrasonography
MRI	Magnetic Resonance Imaging
CT	Computer Tomography
bFGF	basic fibroblast growth factor
